# Insights into the in vitro biological properties of Australian beach‐cast brown seaweed phenolics

**DOI:** 10.1002/fsn3.4415

**Published:** 2024-09-15

**Authors:** Vigasini Subbiah, Faezeh Ebrahimi, Xinyu Duan, Osman Tuncay Agar, Colin J. Barrow, Hafiz A. R. Suleria

**Affiliations:** ^1^ Centre for Sustainable Bioproducts Deakin University Waurn Ponds Victoria Australia; ^2^ School of Agriculture, Food and Ecosystem Sciences, Faculty of Science The University of Melbourne Parkville Victoria Australia

**Keywords:** anti‐diabetic effect, anti‐inflammatory effect, anti‐mitotic activity, phenolics, seaweeds

## Abstract

Five Australian seaweed species, *Phyllosphora comosa*, *Ecklonia radiata*, *Durvillaea potatorum*, *Sargassum fallax*, and *Cystophora siliquosa*, thrive along the country's shorelines. Some of these seaweeds have recognized health benefits but have not been fully investigated in terms of their bioactive components and mechanisms of action. We employed ultrasonication with 70% methanol to extract phenolic compounds from these seaweeds and investigated a range of bioactivities for these extracts, including anti‐inflammatory activity exploring urease inhibition, nitric oxide scavenging activity, protein denaturation inhibition, and protease inhibition. Anti‐diabetic activities were investigated using α‐amylase and α‐glucosidase inhibition assays. Anti‐proliferative and anti‐mitotic activities were evaluated using yeast‐cell and green‐gram models, respectively. Our findings showed that *C. siliquosa* inhibited nitric oxide, urease, and protease activities, with *S. fallax*, *P. comosa*, and *E. radiata* exhibiting substantial inhibition of protein denaturation. *E. radiata* displayed inhibitory effects on both α‐amylase and α‐glucosidase, whereas *P. comosa* targeted only the α‐glucosidase enzyme, indicating different mechanisms of anti‐diabetic activity. In these anti‐mitotic assays, *C. siliquosa* exhibited low cell viability and a significant anti‐proliferative effect, particularly within 24 h, while *E. radiata* demonstrated notable inhibition at 48 h. LC‐ESI‐QTOF‐MS/MS investigation identified 48 phenolic compounds, including 19 phenolic acids, 20 flavonoids, and 9 other polyphenols. The presence of these compounds in extracts correlated with observed biological activities. These results support the potential health benefits of these seaweeds and link this activity to the presence of bioactive phenolics.

## Graphical abstract

Five Australian beach‐cast seaweed phenolics exhibited diverse bioactive properties. *Cystophora siliquosa* inhibited nitric oxide, urease, and protease activities, and showed significant anti‐mitotic activity at 24 h. *Ecklonia radiata* inhibited both α‐amylase and α‐glucosidase enzymes and demonstrated mitotic inhibition at 48 h. Using 70% methanol extraction, 12, 10, 7, 19, and 23 phenolic compounds were identified in *Phyllosphora comosa*, *Ecklonia radiata*, *Durvillaea potatorum*, *Sargassum fallax*, and *Cystophora siliquosa*, respectively.

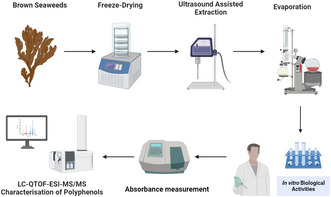

## 1 INTRODUCTION

There has been an increasing focus on the potential of macroalgae as food and as bioactive leads for new pharmaceuticals, partly due to relatively unexplored marine diversity versus the terrestrial counterpart (Tenorio‐Rodríguez et al., 2019). Schepers et al. (2020) and others have shown that the marine environment, including marine algae, contains an array of chemical diversity that is different from terrestrial plants. Numerous studies have demonstrated anti‐inflammatory, anti‐diabetic, anti‐mitotic, anti‐proliferative, and protease inhibition properties of marine seaweed extracts (Hell et al., 2017; Tanna & Mishra, 2019). Phenolic compounds found in seaweed, such as gallic acid, phlorotannins, flavonoids, and phenolic terpenoids, contribute to their positive health benefits and are associated with a range of bioactivities including anti‐inflammatory and anti‐diabetic activities (Lomartire et al., 2021). Seaweeds have been a traditional staple in Asian cuisine for centuries including China, Japan, Korea, the Philippines, and Indonesia (Birch et al., 2019), and their popularity is on the rise in Western countries (Kumar et al., 2008) due to their recognized health benefits (Schepers et al., 2020; Tanna & Mishra, 2019).

Seaweeds are known to have anti‐inflammatory activity and contain compounds that could offer alternatives to commercial anti‐inflammatory drugs like aspirin, ibuprofen, and diclofenac, which often come with side effects such as gastrointestinal ulceration (Rabecca et al., 2022). Study by Kazlowska et al. (2010) reported that *Porphyra dentata* was observed to reduce nitric oxide production. Similarly, another study observed that the *Sargassum duplicatum* reduced the production of nitric oxide (Jaswir et al., 2014). Furthermore, seaweeds exhibit promising anti‐diabetic properties, providing a potential to assist in managing conditions like diabetes mellitus (Shafay et al., 2022). Seaweeds have shown anti‐proliferative effects and anti‐mitotic activity, indicating potential utility in cancer treatment or prevention. For instance, *Ecklonia* spp. displayed a moderate anti‐proliferative effect against breast cell lines, with specific phenolic compounds like dieckol and phloroglucinol demonstrating inhibitory effects on proliferation (Organization, 2022; Rocha et al., 2018).

Phenolic compounds are identified and characterized through liquid chromatography coupled with electrospray‐ionization quadrupole time‐of‐flight mass spectrometry (LC‐ESI‐QTOF‐MS/MS). Brown seaweed has high levels of phenolic compounds, including gallic acid, 2‐hydroxybenzoic acid, 2,3‐dihydroxybenzoic acid, caffeic acid, sinapic acid, and catechin (Subbiah, Duan, et al., 2023a; Subbiah, Ebrahimi, et al., 2023b). In our recent study, we explored the biological properties of various edible brown Australian seaweeds through a series of in vitro assays. The investigated species included *Phyllosphora comosa*, *Ecklonia radiata*, *Durvillaea potatorum*, *Sargassum fallax*, and *Cytosphora siliquosa*, which are abundant along the Australian coastlines. The focus of our investigation centered on the phenolic extract of these seaweeds, with a comprehensive assessment that included urease inhibition, nitric oxide radical scavenging, protein denaturation inhibition, protease inhibition, anti‐proliferative effects, mitotic activity inhibition, and anti‐diabetic properties. After these in vitro assays, we conducted an LC‐ESI‐QTOF‐MS/MS study to characterize and identify the phenolic compounds present. This comparison aimed to ascertain whether the identified compounds correlated with the observed biological effects in the in vitro assays.

## 2 MATERIALS AND METHODS

### 2.1 Sample collection and preparation

Seaweed samples, including *Phyllospora comosa*, *Ecklonia radiata*, *Durvillaea potatorum*, *Sargassum fallax*, and *Cystophora siliquosa*, were gathered from Queenscliff Harbor in Victoria, Australia (38°15′54.0″ S 144°40′10.3″ E). Species identification was performed at the Deakin Marine Institute in Queenscliff and at The University of Melbourne in Parkville. After collection, the seaweed was washed with tap water and Milli‐Q water to remove any extraneous impurities. Subsequently, the samples were manually cut into 1–3 cm pieces using a stainless‐steel, food‐grade knife. After freezing at −70°C for 24 h in a Thermo scientific freezer, the samples were further dried for 72 h at −60°C in a freezer dryer, following the procedure outlined by Subbiah, Duan, et al. (2023a). The resulting frozen seaweed was ground into a fine coarse powder using a grinder (Cuisinart Nut and Spice grinder 46,302, Melbourne, VIC) and stored in a cold room. The dried samples were extracted with 70% methanol using an ultrasonication methodology. The methanol solvent in the extract was removed by rotary evaporation. Subsequently, the samples were freeze‐dried at −60°C for 72 h. Later, the freeze‐dried seaweed powder was mixed with MiliQ water at 1 mg/mL for further analysis.

### 2.2 Chemicals and reagents

Analytical grade standards and chemicals used in this study were purchased from Sigma‐Aldrich Chemicals (Castle Hill, NSW, Australia). The chemicals utilized in this study were urea, active chloride, sodium hydroxide, sodium nitroprusside, phenol, bovine serum albumin, tris (hydroxymethyl) aminomethane, hydrochloric acid, sodium phosphate buffer, calcium chloride, phosphate‐buffered saline, trichloroacetic acid, Griess reagents, and glucose, purchased from Sigma‐Aldrich Chemicals (Castle Hill, NSW, Australia). Trypsin and jack bean urease enzymes were bought from Sigma‐Aldrich Chemicals (Castle Hill, NSW, Australia). Fresh eggs, yeast, green‐gram seeds, and potatoes were bought from the supermarket in Melbourne, Australia. The standards including thiourea, quercetin, ibuprofen, doxorubicin, and diclofenac sodium were purchased from Sigma‐Aldrich Chemicals (Castle Hill, NSW, Australia).

### 2.3 Anti‐inflammatory activity

#### 2.3.1 Urease inhibition assay

The urease activity was quantified using a modified version of Weatherburn's indophenol method, as described by Rani et al. (2022). The assay assessed urease activity by quantifying ammonia production in the reaction mixture. In a 96‐well plate, 25 μL of jack bean urease enzyme (4 U/mL) was combined with 50 μL of buffer (100 mM urea) and 10 μL of seaweed samples. The reaction mixture underwent a 15 min incubation period. Subsequently, 70 μL of alkali (0.1% active chloride (NaOCl) and 0.5% sodium hydroxide (NaOH) w/v) and 45 μL of phenol reagents (0.005% w/v sodium nitroprusside and 1% w/v phenol) were introduced to the reaction mixture. The resulting mixture was further incubated for 50 min, and the absorbance was read at 630 nm using a microplate reader (Thermo Fisher Scientific, Waltham, MA). Thiourea served as the standard in this study due to its role as a urease inhibitor.

The % inhibition was calculated using the following equation:

$$\text{Percentage inhibitions}\;\left(\%\right)=100–\left(A_{t}/A_{c}\right)\times 100;$$

where *A*_t_ is the absorbance of the sample and *A*_c_ is the absorbance of the control.

#### 2.3.2 Nitric oxide radical scavenging activity

The nitric oxide (NO) scavenging activity was assessed using a method adapted from Kumar et al. (2020). In this assay, 2 mL of sodium nitroprusside (5 mM) in standard phosphate buffer saline (0.025 mM, pH 7.4) was mixed with 0.5 mL of seaweed extracts at various concentrations and incubated for 3 h. A control sample does not contain the test compounds instead added an equivalent amount of buffer. Following the 3 h incubation, the samples were diluted with 1 mL of Griess reagents and incubated for an additional 30 min. The entire experiment was conducted under light, and the resulting color formed a purple azo dye. The absorbance of this color was then measured at 550 nm using a spectrophotometer. Ascorbic acid was used as the standard in this assay.

The percentage of nitric oxide inhibition was calculated using the formula:

$$\mathrm{NO}\%=\left(A_{c}–A_{t}\right)/A_{c}\times 100;$$

where *A*_t_ is the absorbance of the test well and *A*_c_ is the absorbance of the control.

#### 2.3.3 Inhibition of protein denaturation

Evaluation of protein denaturation inhibition was conducted following a modification of Dharmadeva et al.'s (2018) protocol. The reaction mixtures were prepared by gently combining 2.8 mL of phosphate‐buffered saline (pH 6.4), 0.2 mL of egg albumin (derived from fresh hen's egg), and 2 mL of seaweed extract. Distilled water was used as a negative control. The resulting mixture underwent incubation in a water bath at 37°C for 20 min, followed by heating at 70°C for an additional 5 min. Subsequently, the reaction mixture was allowed to cool down at room temperature for 15 min. The absorbance of the reaction mixture was then measured at 680 nm using a spectrophotometer. The experiment was conducted in triplicates for each sample, and the absorbance values were recorded. Ibuprofen served as the standard in this assay.

The percentage of inhibition was calculated based on the formula:

$$\%\text{inhibition}=\left(1-A_{c}/A_{t}\right)\times 100;$$

where *A*_c_ is the absorbance of control and *A*_t_ is the absorbance of the test.

### 2.4 Inhibition of protease assay

The assay, adapted from Modi et al. (2019), Thapa and Bajracharya (2014), and Sarveswaran and Suresh (2017), involved combining 0.06 mL of trypsin (1 mg/mL), 1 mL of 20 mM Tris–HCl buffer (pH 7.4), and 1 mL of seaweed samples or the standard in the reaction mixture. This mixture underwent an initial incubation for 10 min at 37°C. Subsequently, 1 mL of 4% bovine serum albumin was introduced, and the mixture was re‐incubated for an additional 20 min at 37°C. After incubation, 2 mL of 5% trichloroacetic acid was added to halt the reaction. The resulting cloudy suspension was then centrifuged at 6729 × *g* for 15 min, and the absorbance of the supernatant was measured at 280 nm. Tris–HCl buffer was utilized as a blank in this experiment, and quercetin was used as a positive control in this experiment which was conducted in triplicate.

The anti‐inflammatory activity was measured by the formula:

$$\%\text{inhibition}=\left(1-A_{c}/A_{t}\right)\times 100;$$

where *A*_c_ is the absorbance of control *A*_t_ is the absorbance of the test.

### 2.5 Antiproliferative assay

The antiproliferative activity was determined using a yeast cell model and the protocol was followed according to Raheel et al. (2017).

Preparation of yeast inoculum involved introducing 5 g of commercially available yeast to 100 mL of sterilized nutrient broth in a conical flask, which was then incubated at 37°C for 24 h. From this seeded broth, 1 mL was diluted with sterilized distilled water to a final volume of 10 mL, resulting in approximately 25.4 × 10^4^ cells.

For the preparation of potato dextrose broth, 200 g of sliced potatoes were boiled for 1 h in 1 L of distilled water, and the resulting filtrate was diluted to 1000 mL using distilled water. Subsequently, 20 g of glucose was added, and the medium was autoclaved.

Cell viability was assessed by combining 2.5 mL of potato dextrose broth (PDB) with 0.5 mL of yeast inoculum and 1 mL of each extract dilution in a test tube. Quercetin and potato dextrose broth containing yeast served as the standard and control, respectively. All test tubes were then incubated at 37°C for 24 h. After incubation, 0.1% methylene blue was mixed with the cell suspension in each sample and examined under low power (10×) of a microscope. The number of living cells (transparent and unstained) and dead cells (stained blue) were counted in 9 chambers of the hemocytometer, and the mean was determined for both the standard and seaweed extract samples.

The cell viability (%) was determined by using the formula:

Percentage of cell viability=Total viable cells/Total cells×100

### 2.6 Anti‐mitotic activity

#### 2.6.1 Seed germination assay

*Vigna radiata* L. (Green‐gram; Family: *Fabaceae*) served as the plant model for this experiment. High‐quality and undamaged *V. radiata* L. seeds were procured from the local market in Melbourne, Australia. Doxorubicin (2 mg/mL) was utilized as the standard drug, while distilled water functioned as the control.

The germination test for *V. radiata* L. seeds adhered to the guidelines outlined in the 2018 International Rules for Seed Testing (ISTA) (Luk Bahadur Chetry, 2018). In a 24‐well microplate, 1 mg/mL concentrations of seaweed phenolic extracts from *P. comosa*, *E. radiata*, *D. potatorum*, *S. fallax*, and *C. siliquosa* were placed. Equal‐weighted *V. radiata* L. seeds were introduced into each well and 500 μL of the sample/ standard were added to each well. The microplate was covered and allowed to germinate at room temperature. A group treated with doxorubicin (at a specified concentration) served as the positive control, while a microplate containing tap water alone was the negative control. The doxorubicin dosage was determined based on a prior study (Murthy et al., 2011). Seed germination was weighed at 24 and 48 h intervals in both the control and test groups. The weight of the seeds was recorded, and the percentage of inhibition was calculated.

The formula used in the calculation of the percentage of inhibition was:

$$\%\text{inhibition}=\left[\left({\mathrm{wt}}_{D}-{\mathrm{wt}}_{E}\right)\right]/\left[\left({\mathrm{wt}}_{D}\right)\right]\times 100$$

where wt_D_ = Seed weight in distilled water; wt_E_ = Seed weight in extract sample; wt_Doxo_ = Seed weight in doxorubicin.

### 2.7 Anti‐diabetic assay

#### 2.7.1 α‐Amylase inhibition assay

The assessment of α‐amylase inhibition by seaweed phenolic extract followed a modified protocol using the chromogenic substrate *p*‐nitrophenyl‐α‐D‐maltohexaoside (PNPG6), as outlined by Wu et al. (2023). A 125 units/mL enzyme working solution was prepared by combining porcine pancreas α‐amylase with sodium phosphate buffer‐I (20 mM, 7 mM sodium chloride (NaCl), 1 mM calcium chloride (CaCl_2_), pH 6.8). Additionally, a 10 mM PNPG6 solution was created in sodium phosphate buffer‐II (20 mM, 7 mM NaCl, pH 6.8). All enzymes and reagents solutions were maintained at a cold temperature in an ice water bath during the preparation. Subsequently, 20 μL of the sample, 100 μL of sodium phosphate buffer‐II, and 50 μL of the enzyme working solution were added to a 96‐well plate. The reaction was initiated by injecting 50 μL of PNPG6 solution and incubated at 37°C for 40 min incubation and the absorbance was read at 410 nm.

The inhibition of α‐amylase was calculated as:

$$\%\text{of inhibition}=\left[1–\left(\left(A_{s}–A_{\mathrm{sb}}\right)/\left(A_{c}–A_{\mathrm{cb}}\right)\right)\right]\times 100;$$

where: *A*_s_ = sample + enzyme + substrate; *A*_sb_ = sample + enzyme + substrate solvent, *A*_c_ = sample solvent + enzyme + substrate, and *A*_cb_ = sample solvent + enzyme + substrate solvent. Acarbose was used as a reference. The results were the mean values of triplicate analyses (*n* = 3). Acarbose was used as a reference.

#### 2.7.2 Glucosidase inhibition assay

The assessment of α‐glucosidase inhibition for seaweed phenolic extract followed the protocol established by Wu et al. (2023), utilizing a mammalian α‐glucosidase enzyme solution prepared from rat intestinal acetone powder. The reaction mixture containing 2 g of rat intestinal acetone powder and 25 mL of potassium phosphate buffer‐I (0.12 M, 1.0% NaCl, pH 6.8) were sonicated in an ice water bath for 5 min at 50 Hz using a Q55 sonicator (Qsonica, CT). The sonicated mixture was then centrifuged for 15 min (18,000 *g*, 4°C), and the supernatant was collected as the α‐glucosidase working solution. The protein content of this solution was determined to be 2.714 mg/mL through a Bradford assay, utilizing bovine serum albumin as a protein standard. Following this, 20 μL of seaweed phenolic extract, 140 μL of potassium phosphate buffer‐II (0.12 M, pH 6.8), and 25 μL of the enzyme working solution were added to a 96‐well plate. The reaction was initiated by introducing 20 μL of 25 mM *p*‐nitrophenyl‐α‐d‐glucopyranoside (PNPG) solution as a substrate, and the mixture was incubated for 60 min at 37°C. The absorbance was measured at 410 nm, and the α‐glucosidase inhibitory activity was calculated using the same formula as the calculation for α‐amylase inhibitory activity. The reported results represent the mean values of three independent analyses (*n* = 3), with acarbose serving as the reference.

### 2.8 Identification and characterization of phenolic compounds

For comprehensive phenolic profiling of seaweeds, liquid chromatography‐electrospray ionization‐quadrupole time‐of‐flight mass spectrometry (LC‐ESI‐QTOF‐MS/MS) was performed using an Agilent 1200 series HPLC system paired with an Agilent 6520 Accurate‐Mass Q‐TOF LC–MS, featuring an electrospray ionization source (ESI), as per a previously established method (Subbiah, Duan, et al., 2023a). The phenolic extracts were filtered through a 0.45 μm syringe filter (Thermo Fisher Scientific Inc., Waltham, MA). Chromatographic separation was achieved using a Synergi Hydro‐RP 80 Å LC Column (250 mm × 4.6 mm, 4 μm, Phenomenex, Torrance, CA) at room temperature, with the sample temperature maintained at 10°C. A 20 μL sample was injected. The binary solvent system consisted of mobile phase A, 99.9% MilliQ water with 0.1% formic acid, and mobile phase B, acetonitrile/MilliQ water/formic acid (95:4.9:0.1), with a flow rate of 0.3 mL/min. The gradient procedure was: 0–2 min hold at 2% B, 2–5 min 2–5% B, 5–25 min 5–45% B, 25–26 min 45–100% B, 26–29 min hold at 100% B, 29–30 min 100–2% B, and 30–35 min hold at 2% B for HPLC equilibration. Peak identification was conducted in both positive and negative modes. Nitrogen gas was used as both the nebulizer and drying gas at 45 psi, with a flow rate of 5 L/min at 300°C. The capillary voltage was set at 3.5 kV, and the nozzle voltage was at 500 V. Mass spectra were obtained in the range of 50–1300 amu. Additionally, MS/MS analyses were performed in automatic mode with collision energies of 10, 15, and 30 eV for fragmentation. Phenolic compounds were analyzed in both positive and negative modes using the LC−ESI−QTOF−MS/MS method, with data processed through Agilent LC–MS Qualitative Software and the Personal Compound Database and Library (PCDL). To ensure high accuracy, compounds were selected for further MS/MS identification and *m/z* characterization only if they had a mass error within ±5 ppm and a PCDL library score above 80%.

### 2.9 Statistical analysis

Statistical analyses and graphical representations were carried out using Minitab 19 (Minitab® for Windows Release 19, Minitab Inc., Chicago, IL), BioRender (Created with BioRender.com), and GraphPad Prism (Version 9.0 for Windows, GraphPad Software, La Jolla, CA). To identify significant differences among samples, a one‐way analysis of variance (ANOVA) was conducted, followed by Tukey's honest significant differences (HSD) test. All assays were conducted in triplicates (*n* = 3).

## 3 RESULTS AND DISCUSSION

### 3.1 Anti‐inflammatory assays

**FIGURE 1:**
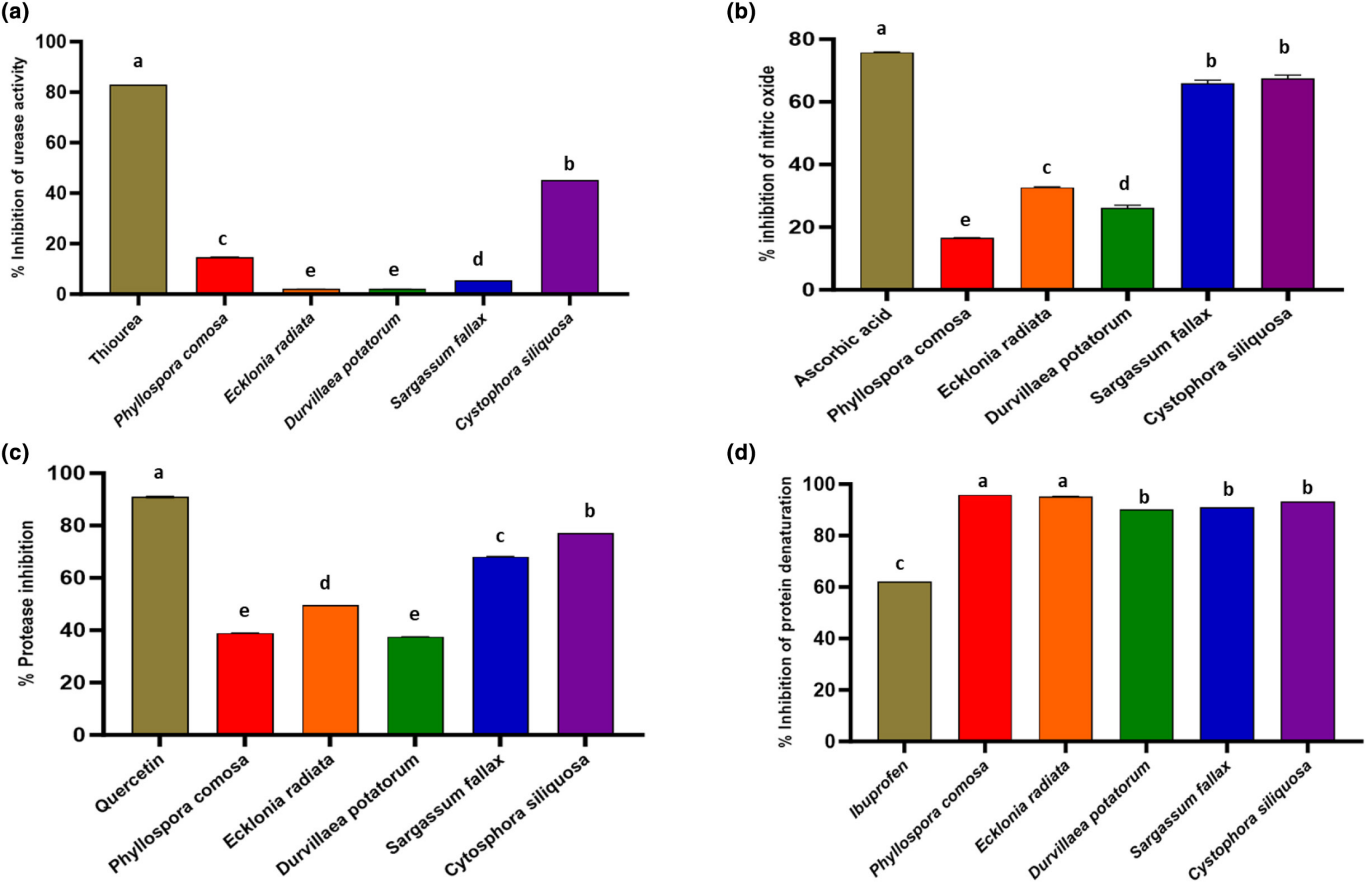
Anti‐inflammatory assay of the seaweed phenolic extract. (a) Urease inhibition assay. (b) Nitric oxide inhibition assay. (c) Protease inhibition assay. (d) Protein denaturation inhibition assay.

The seaweeds investigated in this study were generally good sources of anti‐inflammatory compounds, with activity shown in Figure 1 for the percentage of inhibition of urease, nitric oxide, protease, and protein denaturation, for the seaweed species *Cytosphora siliquosa*, *Phyllospora comosa*, *Sargassum fallax*, *Ecklonia radiata*, and *Durvillaea potatorum* (Ochrophyta, Phaeophyceae).

Urease, an enzyme responsible for converting urea to ammonia and carbon dioxide, is implicated in creating an acidic environment by *Helicobacter pylori* bacteria, leading to gastrointestinal diseases, urinary stones, and pyelonephritis (Mahernia et al., 2015). Therefore, inhibitors of urease hold significant potential in eliminating infections caused by bacteria that produce urease (Amin et al., 2013). Our study observed significant variations with extract from *C. siliquosa* (45.21%) showing higher inhibition than *P. comosa* (14.66%) and *S. fallax* (5.37%), compared with the positive control, thiourea, that has inhibition of 83.02%. Inhibition in this assay was very low for *Ecklonia radiata* and *D. potatorum* extracts.

The results of scavenging of NO radicals in the brown seaweed extracts are expressed as a percentage of scavenging, compared to the positive control ascorbic acid (Figure 1). Among the seaweeds, *C. siliquosa* and *S. fallax* had the highest nitric oxide inhibition at the maximum concentration of 1 mg/mL. Nitric oxide, an essential chemical mediator produced by endothelial cells, macrophages, and neurons, plays a role in regulating diverse physiological processes (Parul et al., 2013). However, elevated levels of nitric oxide are generated and formed only during elevated inflammation in the human body (Jagetia & Baliga, 2004). We can observe in our data that seaweed extracts can stop the series of reactions initiated by excess generation of nitric oxide. A previous study reported that the brown seaweed *Padina boergesennii* exhibited 64.80% inhibition of nitric oxide at a similar concentration (Palanisamy Senthil Kumar, 2012). In another study, *E. radiata* and *P. comosa* showed significantly high nitric oxide inhibition activity (McCauley et al., 2014).

Proteases constitute approximately 2% of the human genome, encompassing 500–600 identified proteases (Vergnolle, 2016). These enzymes catalyze the hydrolysis of peptide bonds, shaping the primary structures of proteins (Leung et al., 2000), and are pivotal in modifying proteins. Proteases play crucial roles in various processes, including digestion, and contribute to defense mechanisms such as blood clotting (García‐Carreño, 1992). Some proteases have been implicated in tumor growth and progression, both in primary and metastatic sites (Eatemadi et al., 2017). In our study, quercetin served as a positive control for comparison with seaweed species for protease activity, as quercetin is a well‐known phenolic compound and an approved nutraceutical. Among the seaweed extracts, *C. siliquosa* demonstrated the highest inhibition, followed by *S. fallax*, *E. radiata*, *P. comosa*, and *D. potatorum*, as shown in Figure 1.

Protein denaturation, induced by external factors like strong acids, bases, heat, or concentrated inorganic salts, leads to alterations in electrostatic hydrogen, hydrophobic, and disulfide bonding in proteins (Kpemissi et al., 2023). This denaturation process, while implicated in conditions such as cancer, rheumatic arthritis, and diabetes, resulting in the production of autoantigens, is also associated with inflammation. Nonsteroidal anti‐inflammatory drugs (NSAIDs) are commonly prescribed to counteract protein denaturation, but they come with potential side effects like autoimmune diseases and gastric ulcers. The egg albumin method offers a cost‐effective alternative for initial detection of anti‐inflammatory activity of seaweed extracts through the denaturation technique (Dharmadeva et al., 2018). In our study, seaweed demonstrated higher protein denaturation inhibition compared to Ibuprofen at a concentration of 1 mg/mL. Specifically, *E. radiata* in our investigation exhibited an impressive 95% inhibition of protein denaturation (Figure 1), surpassing a previous study that reported 63.9% inhibition at the same concentration (Saibu et al., 2023).

### 3.2 In vitro anti‐cancer activity

#### 3.2.1 Anti‐proliferation

The yeast model is a widely utilized approach for studying anti‐cancer activities through antiproliferative assays. Yeast cells share a significant degree of sequence and functional similarity with human cells, making them valuable for investigating biological pathways relevant to both yeast and humans. These pathways include those involved in cell cycle control and DNA damage repair (Raheel et al., 2017). In our study, the anti‐proliferation activity of seaweed extract was assessed using yeast cells, as shown in Table 1. Among the seaweed species, *E. radiata* and *S. fallax* demonstrated the highest antiproliferative effect.

**TABLE 1:** Biological properties of seaweed phenolic extract.

Biological properties	*Phyllosphora comosa*	*Ecklonia radiata*	*Durvillaea potatorum*	*Sargassum fallax*	*Cystophora siliquosa*	Positive standards
Anti‐proliferation (%)	53.58 ± 4.68^b^	61.31 ± 4.05^a^	47.02 ± 0.9^c^	61.16 ± 0.13^a^	54.5 ± 0.43^b^	61.79 ± 0.35^a^ (Quercetin)
Anti‐mitotic activity (%) (24 h)	1.81 ± 2.82^d^	2.96 ± 4.42^cd^	1.72 ± 1.09^d^	3.76 ± 3.61^c^	5.46 ± 3.41^b^	56.46 ± 0.01^a^ (Doxorubicin)
Anti‐mitotic activity (%) (48 h)	—	6.09 ± 2.02^b^	—	2.30 ± 2.94^c^	2.13 ± 1.03^c^	60.94 ± 0.01^a^ (Doxorubicin)
α‐Amylase inhibition (%)	—	12.18 ± 0.01^b^	—	—	—	90.29 ± 0.01^a^ (Acarbose)
α‐Glucosidase inhibition (%)	5.13 ± 0.01^b^	17.55 ± 0.01^b^	—	—	—	72.06 ± 0.01^a^ (Acarbose)

*Note*: All values are expressed as the percentage mean ± standard deviation (*n* = 3). Alphabetic letters indicate significant differences (*p* < .05) in a row using a one‐way analysis of variance (ANOVA) and Tukey's test.

#### 3.2.2 Anti‐mitotic activity

In this assay, the inhibition of cell division was assessed using a green‐gram seed model. The standard drug doxorubicin exhibited the highest inhibition rate, recording 56.46% and 60.94% at 24 and 48 h, respectively. Among the seaweed extracts, *C. siliquosa* demonstrated the highest inhibition, reaching 5.46% at a concentration of 1 mg/mL after 24 h, followed by *S. fallax* with an inhibition rate of 3.76%. Additionally, *E. radiata* exhibits inhibition of 6.09% at 48 h (Table 1). A previous study reported that many chemotherapeutic drugs exert their effects by disrupting cell division (mitosis) in rapidly dividing cells (Jose et al., 2015).

### 3.3 Anti‐diabetic activity

In the anti‐diabetic study, α‐amylase and α‐glucosidase inhibitions are shown in Table 1. α‐Amylase is one of the important enzymes involved in breaking down long‐chain carbohydrates and α‐amylase inhibitors are potential targets in the development of treatment of diabetics. In our study, only the seaweed extract *E. radiata* showed inhibition activity (12.18%) with the remaining seaweed phenolic extract showing no inhibition. Lordan et al. (2013) and others have reported that Phaeophyceae seaweeds can be an excellent source of α‐amylase inhibitors. α‐Glucosidase is another important enzyme involved in breaking down starch and disaccharides to glucose. In this study, the seaweed extract of *P. comosa* and *E. radiata* showed inhibition of α‐glucosidase of 5.13% and 17.55%, respectively, with the other seaweed extracts showing no activity.

### 3.4 *LC‐ESI‐QTOF‐MS/MS* characterization of phenolic compounds

Phenolic compounds were qualitatively analyzed using LC‐ESI‐QTOF‐MS/MS in both positive and negative ionization modes. Compounds meeting criteria, including a mass error <±5 ppm and a PCDL library score exceeding 80, were chosen for subsequent identification and *m/z* characterization through MS/MS analysis. In this study, MS/MS analysis was conducted and the number of compounds identified was 48 in 70% methanol extract of seaweeds as shown in Table 2.

**TABLE 2:** Characterization of phenolic compounds in seaweed samples 70% methanol extract by LC‐ESI‐QTOF‐MS/MS.

No.	Proposed compounds	Molecular formula	RT (min)	Ionization (ESI^+^/ESI^−^)	Molecular weight	Theoretical (*m/z*)	Observed (*m/z*)	Error (ppm)	MS^2^ product ions	Seaweed samples
Phenolic acid										
Hydroxybenzoic acids										
1	Protocatechuic acid 4‐*O*‐glucoside	C_13_H_16_O_9_	14.994	[M − H]^−^	316.0786	315.0713	315.0702	−3.5	153	Cyst, Sarg*
2	Gallic acid 4‐*O*‐glucoside	C_13_H_16_O_10_	30.462	[M − H]^−^ **	332.0768	331.0695	331.0691	−1.2	169, 125	Phyl, Sarg*
3	Gallic acid	C_7_H_6_O_5_	31.309	[M − H]^−^	170.0225	169.0152	169.0154	1.2	125	Phyl, Eckl*
4	2‐Hydroxybenzoic acid	C_7_H_6_O_3_	32.019	[M − H]^−^	138.0310	137.0237	137.0238	0.7	93	Sarg
Hydroxycinnamic acids										
5	*m*‐Coumaric acid	C_9_H_8_O_3_	5.228	[M − H]^−^	164.0487	163.0414	163.0412	−1.2	119	Durv, Sarg
6	Caffeoyl tartaric acid	C_13_H_12_O_9_	5.426	[M − H]^−^	312.0504	311.0431	311.0438	2.3	161	Cyst, Sarg, Phyl*
7	Isoferulic acid 3‐sulfate	C_10_H_10_O_7_S	5.608	[M − H]^−^	274.0129	273.0056	273.0054	−0.7	193, 178	Phyl, Eckl*
8	Caffeic acid 3‐*O*‐glucuronide	C_15_H_16_O_10_	6.388	[M − H]^−^ **	356.0743	355.0670	355.0673	0.8	179	Phyl, Cyst*
9	Cinnamic acid	C_9_H_8_O_2_	7.246	[M − H]^−^	148.0537	147.0464	147.0465	0.7	103	Eckl, Durv*
10	1‐Sinapoyl‐2‐feruloylgentiobiose	C_33_H_40_O_18_	14.898	[M − H]^−^ **	724.2205	723.2132	723.2136	0.6	529, 499	Eckl, Durv, Sarg*
11	Sinapic acid	C_11_H_12_O_5_	16.158	[M − H]^−^ **	224.0666	223.0593	223.0595	0.9	205, 163	Sarg*
12	*p*‐Coumaroyl tartaric acid	C_13_H_12_O_8_	16.214	[M − H]^−^	296.0555	295.0482	295.0486	1.4	115	Sarg, Cyst*
13	*p*‐Coumaroyl malic acid	C_13_H_12_O_7_	16.596	[M − H]^−^ **	280.0596	279.0523	279.0512	−3.9	163, 119	Sarg, Cyst*
14	Ferulic acid 4‐*O*‐glucuronide	C_16_H_18_O_10_	17.199	[M − H]^−^ **	370.0895	369.0822	369.0836	3.8	193	Eckl, Cyst*
15	Rosmarinic acid	C_18_H_16_O_8_	18.249	[M − H]^−^	360.0850	359.0777	359.0775	−0.6	179	Sarg, Cyst*
16	Caffeoyl glucose	C_15_H_18_O_9_	18.549	[M − H]^−^	342.0951	341.0878	341.0890	3.5	179, 161	Sarg*
17	5–5′‐Dehydrodiferulic acid	C_20_H_18_O_8_	21.657	[M − H]^+^ **	386.0985	385.0912	385.0913	0.3	369	Cyst*
Hydroxyphenylpropanoic acids										
18	Dihydroferulic acid 4‐*O*‐glucuronide	C_16_H_20_O_10_	3.119	[M − H]^−^ **	372.1090	371.1017	371.1019	0.5	195	Eckl, Cyst*
19	Dihydrocaffeic acid 3‐*O*‐glucuronide	C_15_H_18_O_10_	7.535	[M − H]^−^	358.0913	357.0840	357.0837	−0.8	181	Eckl, Cyst*
Flavonoids										
Flavanols										
20	(+)‐Catechin 3‐*O*‐gallate	C_22_H_18_O_10_	20.261	[M − H]^−^	442.0879	441.0806	441.0811	1.1	289, 169, 125	Cyst*
21	Theaflavin 3,3′‐*O*‐digallate	C_43_H_32_O_20_	24.533	[M − H]^−^	868.1448	867.1375	867.1373	−0.2	715, 563, 545	Eckl*
22	(−)‐Epigallocatechin	C_15_H_14_O_7_	30.714	[M − H]^−^ **	306.0737	305.0664	305.0670	2.0	261, 219	Cyst*
Flavones										
23	Apigenin 6‐C‐glucoside	C_21_H_20_O_10_	16.981	[M − H]^−^	432.1077	431.1004	431.1015	2.6	413, 341, 311	Sarg*
24	Isorhamnetin	C_16_H_12_O_7_	19.215	[M − H]^−^	316.0575	315.0502	315.0510	2.5	300, 271	Sarg
25	Apigenin 7‐*O*‐glucuronide	C_21_H_18_O_11_	22.686	[M − H]_−_ **	446.0875	445.0802	445.0802	0.2	271, 253	Phyl*
Flavanones										
26	Hesperetin 3′,7‐*O*‐diglucuronide	C_28_H_30_O_18_	4.806	[M − H]^−^	654.1424	653.1351	653.1356	0.8	477, 301, 286, 242	Durv, Phyl*
27	Narirutin	C_27_H_32_O_14_	5.264	[M − H]^−^	580.1827	579.1754	579.1756	0.3	271	Durv*
28	Hesperetin 3′‐sulfate	C_16_H_14_O_9_S	7.389	[M − H]^−^	382.0354	381.0281	381.0277	−1.0	301, 286, 257, 242	Phyl, Durv, Sarg*
29	Hesperetin 3′‐*O*‐glucuronide	C_22_H_22_O_12_	13.741	[M − H]^−^ **	478.1130	477.1057	477.1062	1.0	301, 175, 113, 85	Cyst*
Flavonols										
30	Quercetin 3′‐*O*‐glucuronide	C_21_H_18_O_13_	5.185	[M − H]^−^	478.0758	477.0685	477.0679	−1.3	301	Eckl*
31	Myricetin 3‐*O*‐arabinoside	C_20_H_18_O_12_	7.182	[M − H]^−^	450.0821	449.0748	449.0750	0.4	317	Cyst*
32	Quercetin 3‐*O*‐arabinoside	C_20_H_18_O_11_	20.258	[M − H]^−^ **	434.0850	433.0777	433.0796	4.4	301	Sarg*
Dihydroflavonols										
33	Dihydroquercetin	C_15_H_12_O_7_	15.971	[M − H]^−^	304.0591	303.0518	303.0518	0.2	285, 275, 151	Cyst, Phyl*
Isoflavonoids										
34	6″‐*O*‐Acetylglycitin	C_24_H_24_O_11_	5.896	[M + H]^+^	488.1333	489.1406	489.1398	−1.6	285, 270	Phyl*
35	2‐Dehydro‐*O*‐desmethylangolensin	C_15_H_12_O_4_	5.898	[M − H]^−^	256.0751	255.0678	255.0679	0.4	135, 119	Durv*
36	2′‐Hydroxyformononetin	C_16_H_12_O_5_	5.934	[M − H]^−^ **	284.0687	283.0614	283.0619	1.8	270, 229	Cyst, Sarg*
37	Violanone	C_17_H_16_O_6_	5.937	[M − H]^−^	316.0932	315.0859	315.0850	−2.9	300, 285, 135	Durv, Cyst*
38	Sativanone	C_17_H_16_O_5_	16.707	[M − H]^−^ **	300.0987	299.0914	299.0914	0.1	284, 269, 225	Cyst*
39	Genistein 4′,7‐*O*‐diglucuronide	C_27_H_26_O_17_	23.862	[M − H]^−^ **	622.1174	621.1101	621.1122	3.4	269	Cyst
Other polyphenols										
Hydroxycoumarins										
40	Scopoletin	C_10_H_8_O_4_	31.117	[M − H]^−^	192.0419	191.0346	191.0347	0.5	176, 147	Eckl*
Phenolic terpenes										
41	Carnosic acid	C_20_H_28_O_4_	32.549	[M − H]^−^ **	332.2004	331.1931	331.1933	0.6	287, 269	Cyst, Sarg, Eckl, Phyl*
Tyrosols										
42	3,4‐DHPEA‐AC	C_10_H_12_O_4_	24.521	[M − H]^−^	196.0736	195.0663	195.0662	−0.5	135	Phyl
43	3,4‐DHPEA‐EDA	C_17_H_20_O_6_	29.370	[M − H]^−^	320.1270	319.1197	319.1192	−1.6	275, 195	Eckl*
Alkylmethoxyphenols										
44	Equol	C_15_H_14_O_3_	16.915	[M + H]^+^	242.0943	243.1016	243.1019	1.2	255, 211, 197	Cyst*
Other polyphenols										
45	Salvianolic acid C	C_26_H_20_O_10_	16.686	[M − H]^−^	492.1026	491.0953	491.0976	4.7	311, 267, 249	Cyst
Lignans										
46	Todolactol A	C_20_H_24_O_7_	16.242	[M − H]^−^	376.1546	375.1473	375.1469	−1.1	313, 137	Phyl, Sarg*
47	Arctigenin	C_21_H_24_O_6_	26.476	[M − H]^−^	372.1565	371.1492	371.1494	0.5	356, 312, 295	Cyst, Sarg*
48	Secoisolariciresinol‐sesquilignan	C_30_H_38_O_10_	31.907	[M − H]^−^	558.2434	557.2361	557.2387	4.7	539, 521, 509, 361	Phyl

* Compound was detected in more than one seaweed sample. Data presented in this table are from the sample indicated by the asterisk.

** The mode of initial detection and confirms detection in the other mode. Compounds were detected in both negative [M − H]^−^ and positive [M + H]^+^ modes of ionization, but data from only one mode is presented. The abbreviations are Phyl “*Phyllosphora comosa*”; Eckl “*Ecklonia radiata*”; Durv “*Durvillaea potatorum*”; Sarg “*Sargassum fallax*”; Cyst “*Cystophora siliquosa*.”

In this study, a total of 19 phenolic acid compounds were identified, including gallic acid (Compound **3**, [M − H]^−^, *m/z* 169.0154) and 2‐hydroxybenzoic acid (Compound **4**, [M − H]^−^, *m/z* 137.0238). These compounds exhibited product ions at *m/z* 125 and *m/z* 93, indicative of the corresponding loss of CO_2_. Gallic acid and 2‐hydroxybenzoic acid, two of the identified compounds, are associated with positive pharmacological effects, including anti‐diabetic, anti‐cancer, and anti‐inflammatory properties. Previous research has demonstrated that gallic acid is beneficial in addressing neurodegenerative diseases, diabetic retinopathy, thyroid dysfunction, and certain types of cancer. 2‐Hydroxybenzoic acid (2‐HBA) has been found to play a significant role in modulating inflammation and cancer, in part through the inhibition of cyclooxygenase‐2 (COX‐2). This dual functionality enhances the compound's potential as a therapeutic agent with implications for both inflammatory conditions and cancer prevention or treatment. The presence of gallic acid in species like *P. comosa* and *E. radiata* may explain their positive effects observed in in vitro biological assays, particularly in anti‐diabetic and anti‐inflammatory evaluations. However, in *S. fallax*, 2‐hydroxybenzoic acid was present and is known to inhibit both nitric oxide and protease activities.

Compound **1** ([M − H]^−^ *m/z* 315.0702) was identified as protocatechuic acid 4‐*O*‐glucoside, confirmed by the presence of a product ion at *m/z* 153 resulting from the loss of a hexosyl moiety (162 Da) from the precursor molecule (Sun et al., 2010). This compound was previously shown to induce cell death in HepG2 cancerous cell line of the liver. The mechanism involves the stimulation of the c‐Jun N‐terminal kinase (JNK) and p38 subgroups of the mitogen‐activated protein kinase (MAPK) family (Abida Kalsoom Khan et al., 2015). Protocatechuic acid exhibits chemopreventive potential by inhibiting in vitro chemical carcinogenesis and exerting proapoptotic and antiproliferative effects (Kakkar & Bais, 2014). Protocatechuic acid exerts its anti‐inflammatory effects via two primary mechanisms. Firstly, it diminishes the generation of inflammatory mediators such as Interleukin‐6 (IL‐6). Secondly, it lowers the levels of inflammatory genes and proteins by influencing pathways like Nuclear factor kappa‐light‐chain‐enhancer of activated B cells (NF‐κB), MAPKs, and Signal transducer and activator of transcription 3 (STAT3) (Wang et al., 2015). While current studies on protocatechuic acid's anti‐inflammatory activity primarily rely on animal and cell experiments, further investigation is essential to provide more reliable data for potential human disease treatments (Song et al., 2020). Our results indicate that *S. fallax* and *D. potatorum* are rich sources of this compound and it is at least partially responsible for the observed high anti‐inflammatory activities. Quercetin 3′‐*O*‐glucuronide (Compound **30** [M−H]^−^ *m/z* at 477.0679) was characterized by a product ion at *m/z* 301 in the MS^2^ spectrum, attributed to the loss of the glucuronide moiety (176 Da) from the precursor (Castro et al., 2020). Previous analyses of Quercetin 3′‐*O*‐glucuronide have indicated its positive impact on the proliferation of neural cells (Baral et al., 2017). In addition to its neurogenic effects, another study demonstrated the inhibitory activity of this compound against α‐glucosidase. In our anti‐*α*‐glucosidase assay, conducted with *E. radiata* having this compound, we observed similar inhibitory effects, consistent with previous results (Xing et al., 2021).

Scopoletin (compound **40** [M − H]^−^ *m/z* at 191.0347) was identified through the presence of product ions at *m/z* 176 [M − H − CH_3_] and *m/z* 147 [M − H − CO_2_] (Zeng et al., 2018). Scopoletin exhibits a diverse range of pharmacological activities, including antihepatotoxicity, antibacterial, antifungal, antitubercular, anti‐migratory, antihypertensive, antioxidant, antiproliferative, anti‐inflammatory, neurological, antidiabetic, and anti‐hyperuricemic properties (Firmansyah et al., 2021). Scopoletin previously demonstrated a potent anti‐proliferative effect on human umbilical vein endothelial cells, suggesting its potential in inhibiting angiogenesis. This is particularly significant in pathological conditions such as cancer, where the growth of new blood vessels supports the nourishment and progression of tumors (Cai et al., 2013). *E. radiata*, which contains this compound, exhibited a substantial (60.07%) anti‐proliferative effect among yeast cells.

Dihydroferulic acid 4‐*O*‐glucuronide (compound **18**, [M − H]^−^) was tentatively identified with precursor ions at *m/z* 371.1019. The presence of the compound was confirmed due to the loss of glucuronide which produced fragment ions at *m/z* 195 (Sasot et al., 2017). Previously a study has shown that dihydroferulic acid 4‐*O*‐glucuronide has the ability to reduce the production of nitric oxide in human umbilical vein endothelial cells (HUVECs) (Serreli et al., 2021). Similarly in our study we found that *E. radiata* and *C. siliquosa* have high in vitro inhibition capacity of nitric oxide.

The extracts rich in phenolic compounds identified in this study exhibit significant pharmacological potential. While some of these compounds have been previously studied with cell lines, others remain unexplored. Future work is required to isolate specific phenolics and further test their bioactivity.

## 4 CONCLUSION

The findings of the present investigation demonstrate the anti‐inflammatory, anti‐diabetic, and anti‐proliferative potential of phenolic extract of the Australian beach‐cast brown seaweeds *P. comosa*, *E. radiata*, *D. potatorum*, *S. fallax*, and *C. siliquosa*. *C. siliquosa* showed high inhibition of nitric oxide, urease, protease, and proliferative activity. Whereas *P. comosa* inhibited protein denaturation, *E. radiata* inhibited both α‐amylase and α‐glucosidase enzymes. Application of LC‐ESI‐QTOF‐MS/MS identified some known bioactive phenolics. This study indicates that beach‐cast brown seaweeds are a good source of biologically active compounds with potential health benefits.

## AUTHOR CONTRIBUTIONS

**Vigasini Subbiah:** Conceptualization (lead); data curation (lead); formal analysis (lead); investigation (lead); methodology (lead); software (lead); validation (lead); visualization (lead); writing – original draft (lead); writing – review and editing (lead). **Faezeh Ebrahimi:** Writing – review and editing (equal). **Xinyu Duan:** Writing – review and editing (equal). **Osman Tuncay Agar:** Writing – original draft (supporting); writing – review and editing (equal). **Colin J. Barrow:** Funding acquisition (lead); project administration (lead); resources (lead); supervision (lead); writing – original draft (equal); writing – review and editing (equal). **Hafiz A. R. Suleria:** Conceptualization (supporting); data curation (supporting); formal analysis (supporting); funding acquisition (lead); investigation (supporting); methodology (supporting); project administration (lead); resources (lead); software (supporting); supervision (lead); validation (supporting); visualization (supporting); writing – original draft (equal); writing – review and editing (equal).

## FUNDING INFORMATION

This research was funded by the Deakin university under “Deakin University Postgraduate Research Scholarship (DUPRS) scheme,” Deakin DVCR‐funded scholarship supporting Deakin BioFactory research; “Collaborative Research Development Grant” (Grant No. UoM‐21/23) funded by the University of Melbourne; and “Australian Research Council—Discovery Early Career Award” (ARC‐DECRA—DE220100055) funded by the Australian Government.

## CONFLICT OF INTEREST STATEMENT

The authors declare no conflict of interest.

## Data Availability

Data will be made available on request.
